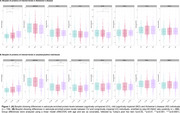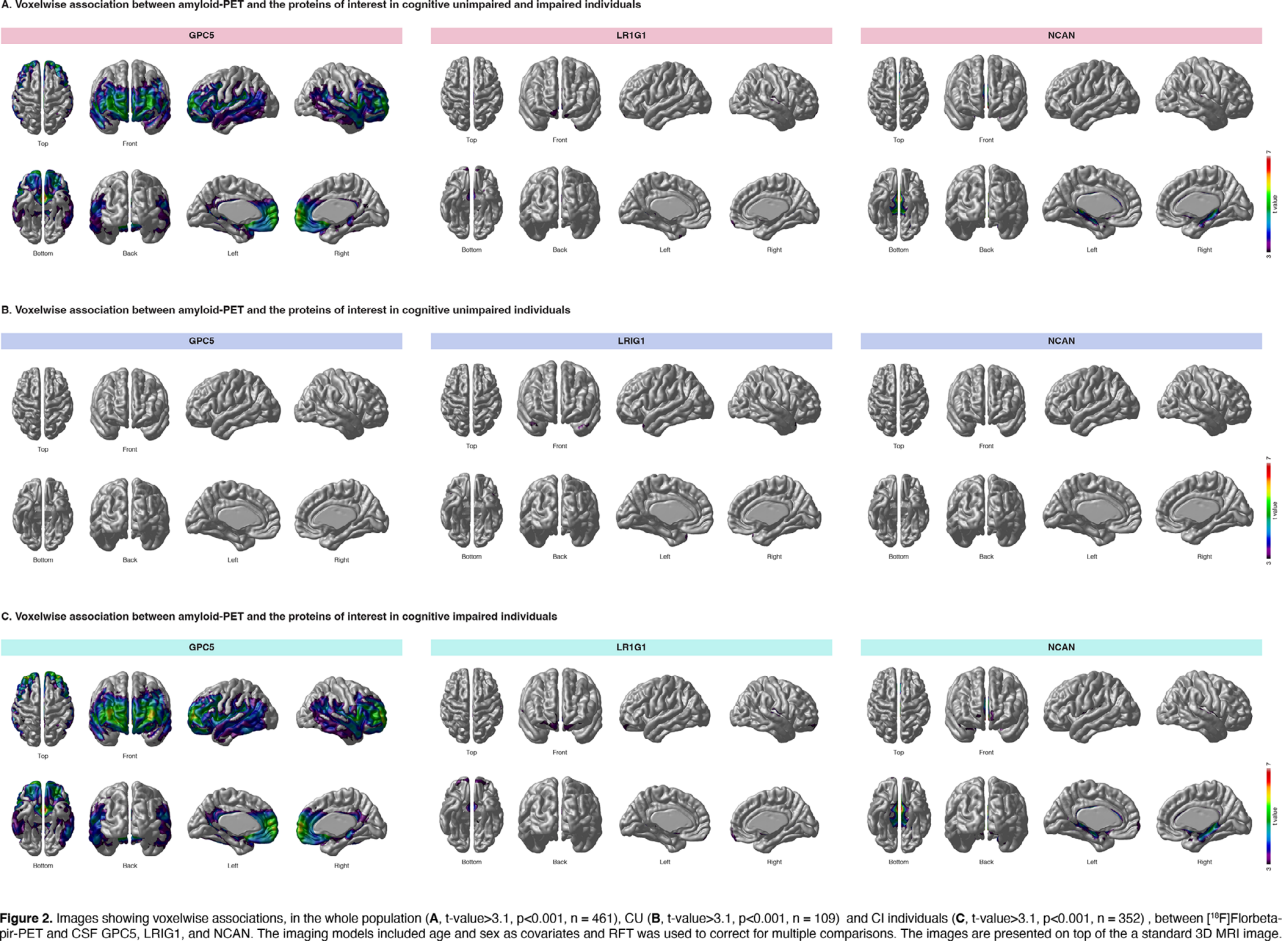# Novel CSF astrocyte biomarkers are associated with amyloid load in Alzheimer’s disease

**DOI:** 10.1002/alz70862_110163

**Published:** 2025-12-23

**Authors:** Luiza Santos Machado, Guilherme Povala, Ilaria Pola, Dzeneta Vizlin‐Hodzic, Pedro Rosa‐Neto, Kaj Blennow, Eduardo R. Zimmer, Henrik Zetterberg, Andrea L. Benedet, Nicholas J. Ashton

**Affiliations:** ^1^ Department of Psychiatry and Neurochemistry, Institute of Neuroscience and Physiology, The Sahlgrenska Academy, University of Gothenburg, Gothenburg, VG Sweden; ^2^ University of Pittsburgh, Pittsburgh, PA USA; ^3^ Department of Psychiatry and Neurochemistry, Institute of Neuroscience and Physiology, The Sahlgrenska Academy, University of Gothenburg, Mölndal Sweden; ^4^ University of Gothenburg, Gothenburg, Västra Götaland Sweden; ^5^ McGill University, Montreal, QC Canada; ^6^ University of Gothenburg, Mölndal Sweden; ^7^ Universidade Federal do Rio Grande do Sul, Porto Alegre, RS Brazil; ^8^ Department of Psychiatry and Neurochemistry, Institute of Neuroscience and Physiology, The Sahlgrenska Academy, University of Gothenburg, Mölndal, Gothenburg Sweden; ^9^ Banner Alzheimer's Institute, Phoenix, AZ USA

## Abstract

**Background:**

Astrocytes are highly involved in Alzheimer’s disease (AD) pathophysiology. GFAP, an astrocyte‐enriched protein, increases in response to amyloid (Aβ) pathology and is used as a fluid biomarker of astrocyte reactivity in AD. However, GFAP does not fully reflect the astrocytic dynamics in response to the disease. Thus, we aimed to identify novel astrocyte biomarkers in CSF that contribute to the understanding of the pathological changes in AD.

**Method:**

We analyzed CSF proteomic data from 728 individuals in the ADNI cohort (SomaLogic). A pre‐defined list of 30 astrocyte‐enriched genes was contrasted with the available ADNI CSF proteomic data, resulting in eight proteins of interest, including GFAP. We examined their CSF levels across cognitively unimpaired (CU), mild cognitively impaired (MCI), and AD individuals (Figure 1a). The proteins levels in CSF were further investigated in CU and cognitively impaired (CI) individuals who were also categorized according to their Aβ status (ptau181/Aβ42 ratio cut‐off=0.028, Figure 1b). Voxelwise models assessed associations between the selected proteins and [^18^F]Florbetapir‐PET, a biomarker of Aβ deposition, in a subset of the individuals (*n* = 461), and CU and CI individuals separately. Models also included age and sex, and RFT was used for multiple comparisons correction in the imaging analyses.

**Result:**

CSF NCAN was significantly reduced in AD individuals compared to CU and MCI (Figure 1a). Further analysis revealed elevated CSF GPC5 levels in Aβ‐positive CI (CI Aβ+) compared to Aβ‐negative CU (CU Aβ‐) and CI (CI Aβ‐) groups. In contrast, CSF LRIG1 and NCAN were only increased in CI+ compared to CI‐ individuals (Figure 1b). Positive associations were observed between CSF GPC5, LRIG1, and NCAN, and [^18^F]Florbetapir‐PET, with GPC5 showing the most widespread cortical associations, particularly in CI individuals (Figure 2).

**Conclusion:**

This study identifies GPC5, LRIG1, and NCAN as CSF astrocyte biomarkers altered across AD cognitive status and amyloid pathology. GPC5, in particular, showed the most widespread cortical association with Aβ deposition, consistent with its role in synaptic maturation and stabilization. Given that GPC5 is highly expressed in cortical astrocytes, these findings highlight its potential as a novel astrocytic biomarker in AD. Further validation will be conducted in an external cohort.